# Plasmon-exciton couplings in the MoS_2_/AuNP plasmonic hybrid structure

**DOI:** 10.1038/s41598-022-26485-4

**Published:** 2022-12-23

**Authors:** Hyuntae Kim, Jaeseung Im, Kiin Nam, Gang Hee Han, Jin Young Park, Sungjae Yoo, MohammadNavid Haddadnezhad, Sungho Park, Woongkyu Park, Jae Sung Ahn, Doojae Park, Mun Seok Jeong, Soobong Choi

**Affiliations:** 1 Department of Physics, Incheon National University, Incheon, 22012 Republic of Korea; 2 Department of Chemistry, Sungkyunkwan University, Suwon, 16419 Republic of Korea; 3 Medical and Bio Photonics Research Center, Korea Photonics Technology Institute (KOPTI), Gwangju, 61007 Republic of Korea; 4 Department of Applied Optics and Physics, Hallym University, Chuncheon, 24252 Republic of Korea; 5 Department of Physics, Hanyang University, Seoul, 04763 Republic of Korea; 6 Department of Energy Engineering, Hanyang University, Seoul, 04763 Republic of Korea

**Keywords:** Nanophotonics and plasmonics, Nanophotonics and plasmonics, Nanophotonics and plasmonics

## Abstract

The understanding and engineering of the plasmon-exciton coupling are necessary to control the innovative optoelectronic device platform. In this study, we investigated the intertwined mechanism of each plasmon-exciton couplings in monolayer molybdenum disulfide (MoS_2_) and plasmonic hybrid structure. The results of absorption, simulation, electrostatics, and emission spectra show that interaction between photoexcited carrier and exciton modes are successfully coupled by energy transfer and exciton recombination processes. Especially, neutral exciton, trion, and biexciton can be selectively enhanced by designing the plasmonic hybrid platform. All of these results imply that there is another degree of freedom to control the individual enhancement of each exciton mode in the development of nano optoelectronic devices.

## Introduction

Optical response in transition metal dichalcogenides (TMDCs) could be engineered by the van der Waals heterostructures, chemical treatment, defect controlling and inducing the local strain, etc^1–6^. Among them, the hybrid structure of noble metal nanoparticles with TMDCs has been introduced to enhance the light-matter interaction caused by plasmon-exciton couplings^7–12^. The techniques for enhancing the optical response are based on the local electromagnetic (EM) field confinements on a metallic nanostructure that is referred to as the localized surface plasmon resonance (LSPR)^13,14^. The localized EM field increases the absorption and emission rate in plasmonic hybrid structures. In addition, the orientation of the hotspots of the EM field could be controlled by designing the plasmonic nanoparticles platform, gap distance, and local environment structure^15–17^.

The molybdenum disulfide (MoS_2_) is one of the noble candidates for functional optical devices due to its unique physical properties, such as tightly bounded excitons, chemical stability, and visible spectral region of photoluminescence (PL) spectra, bandgap tuning by local induced strain^18^. In the previous studies of MoS_2_/plasmonic hybrid structure, these plasmonic nanoparticles are employed in various structures, such as nano-rings, heterodimers, plates, rods, 3D cubes, and split rings, etc^19–21^. Among them, the non-centrosymmetric plasmonic structures have attracted a lot of interest because the possibility of creating and manipulating the hotspot with only single AuNP^21,22^. The mechanism of plasmon-exciton couplings was explained by hot-electron injection, energy transfer, bandgap funneling, and EM field enhancement^23–25^.

The exciton dynamics of photogenerated quasiparticles, which are the neutral excitons(X^0^), trions(X^–^), and biexcitons(XX), are electronically excited states consisting of an electron and a hole. The X^–^ is the formation of the interaction between the bounded electron–hole pair and the additional electron or hole in the opposite valley. The XX is formed by two electron–hole pair in opposite valley. They also can be generated when the material is electrically or optically doped, respectively^26^. The recombination processes of excitons from X^0^ to X^–^ and XX are affected by the background carrier concentrations and are also controlled by Fermi level tunning by local doping^27^. The optical response of XX in MoS_2_ depends on the optical doping. However, the X^0^ and X^–^ do on the electrical doping dependence^26,28–30^. So far as we know, the coupling mechanism of plasmon-exciton has remained vague due to its complex recombination pathways in each exciton mode.

In this paper, we investigate the intertwined mechanism of plasmon-exciton couplings in X^0^, X^–^, and XX modes on the hybrid structure of the monolayer MoS_2_ and non-centrosymmetric Au nanoparticle (AuNP). To characterize the optical response, we measured the absorption, Raman, and PL spectra depending on the excitation wavelength, incident power, and polarization. Also, non-centrosymmetric AuNP opens the possibility to control the plasmon-exciton coupling due to the optical response dependence on the polarization. The power-dependent PL spectra of each exciton mode are used to identify the optical doping effect and charge recombination processes in multiexciton modes.

## Results

**Figure 1:**
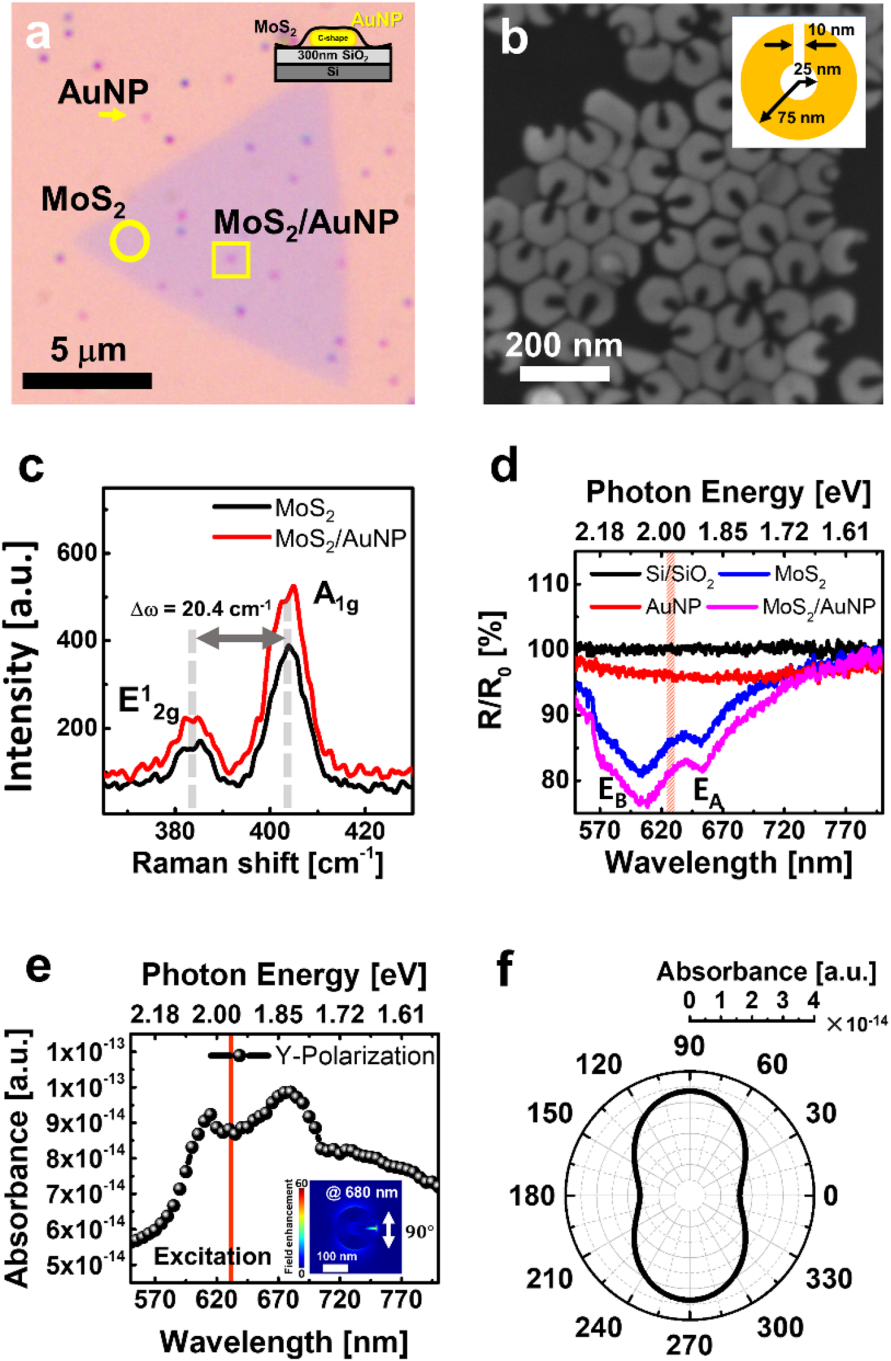
Monolayer MoS_2_/AuNP characterization. (**a**) Optical image of monolayer MoS_2_/AuNP hybrid. The inset is cross-sectional schematics of structure. (**b**) SEM image of non-centrosymmetric AuNP. The inset represents a dimension of AuNP. (**c**) 532 nm excitation Raman scattering spectra. (**d**) The relative reflectance spectra in the 550 ~ 800 nm range. Si/SiO_2_ was set as the reference surface. (**e**) Simulation result of absorbance spectrum on the AuNP, which result set the 90 degrees of incident polarization. The red line indicates the resonance excitation wavelength of 632.8 nm. The inset shows the electric field distribution of AuNP. (**f**) FEM simulation result of polarization dependence absorbance strength.

Figure 1A shows the optical images of the MoS_2_/plasmonic hybrid structures. The spatial density of the AuNPs is 0.13 particles/µm^2^. The indicated regions are the bare MoS_2_ (circle), MoS_2_/AuNP hybrid structure (square), and AuNP particle without MoS_2_ (arrow), respectively. The high-resolution scanning electron microscope (SEM) image shows the geometrical shape of AuNP (Fig. 1b). The AuNPs have an outer radius of ~ 75 nm, an inner radius of ~ 25 nm, and a gap width of ~ 10 nm, respectively (inset of Fig. 1b).

As depicted in Fig. 1c, the Raman spectra of the monolayer MoS_2_ and MoS_2_/AuNP hybrid structure under 532 nm excitations clearly show A_1g_ and E^1^_2g_ peaks. The frequency difference of Raman modes between the E^1^_2g_ and A_1g_ in the monolayer MoS_2_ was observed as 20.4 cm^−1^, which is consistent with the previous studies^31,32^. On the other hand, in the case of the MoS_2_/AuNP hybrid structure, the relative peak intensity ratio between A_1g_ and E^1^_2g_ modes, A_1g_/E^1^_2g_, was increased. It can be explained as a result of the p-doping effect due to the tensile strain of the MoS_2_ surface on the AuNP^33^.

Figure 1d shows the reflectance from Si/SiO_2_, AuNP, MoS_2_, and MoS_2_/AuNP. The Si/SiO_2_ was used as a reference surface. AuNP does not show strong resonant absorption feature, but about 5% decreased reflectance in the 550 nm to 800 nm spectral range because of the LSPRs absorption. The MoS_2_ and MoS_2_/AuNP show two absorption dips at ~ 651 nm (E_A_) and ~ 604 nm (E_B_), which correspond to the absorption band of A and B excitons in MoS_2_, respectively^34,35^. In the MoS_2_/AuNP hybrid structure, the reflectance was additionally decreased (i.e., absorption increased) at E_A_ and E_B_ by 5% than the bare MoS_2_ monolayer. The absorption enhancement originates from the response of LSPRs on the AuNP with the resonant energy transfer^36^. Therefore, the excitation wavelength of 632.8 nm is suitable for enhanced absorption via LSPR.

In addition, the absorbance spectrum of the AuNP was calculated by the finite element method (FEM) simulation, as shown in Fig. 1e,f. The simulated absorbance spectrum has two resonance peaks at 615 nm and 680 nm. In our experiment, two excitation wavelengths of the laser (632.8 nm and 532 nm) were used to compare the on/off resonance characteristics of the MoS_2_/AuNP hybrid structure (Fig. 1e). The AuNPs have polarization-dependent absorption, which originated from their non-centrosymmetric structure^37^. The absorption can be enhanced only when the polarization and the excitation wavelength of the light are satisfied with the resonance condition of the AuNP (Fig. 1f).

**Figure 2:**
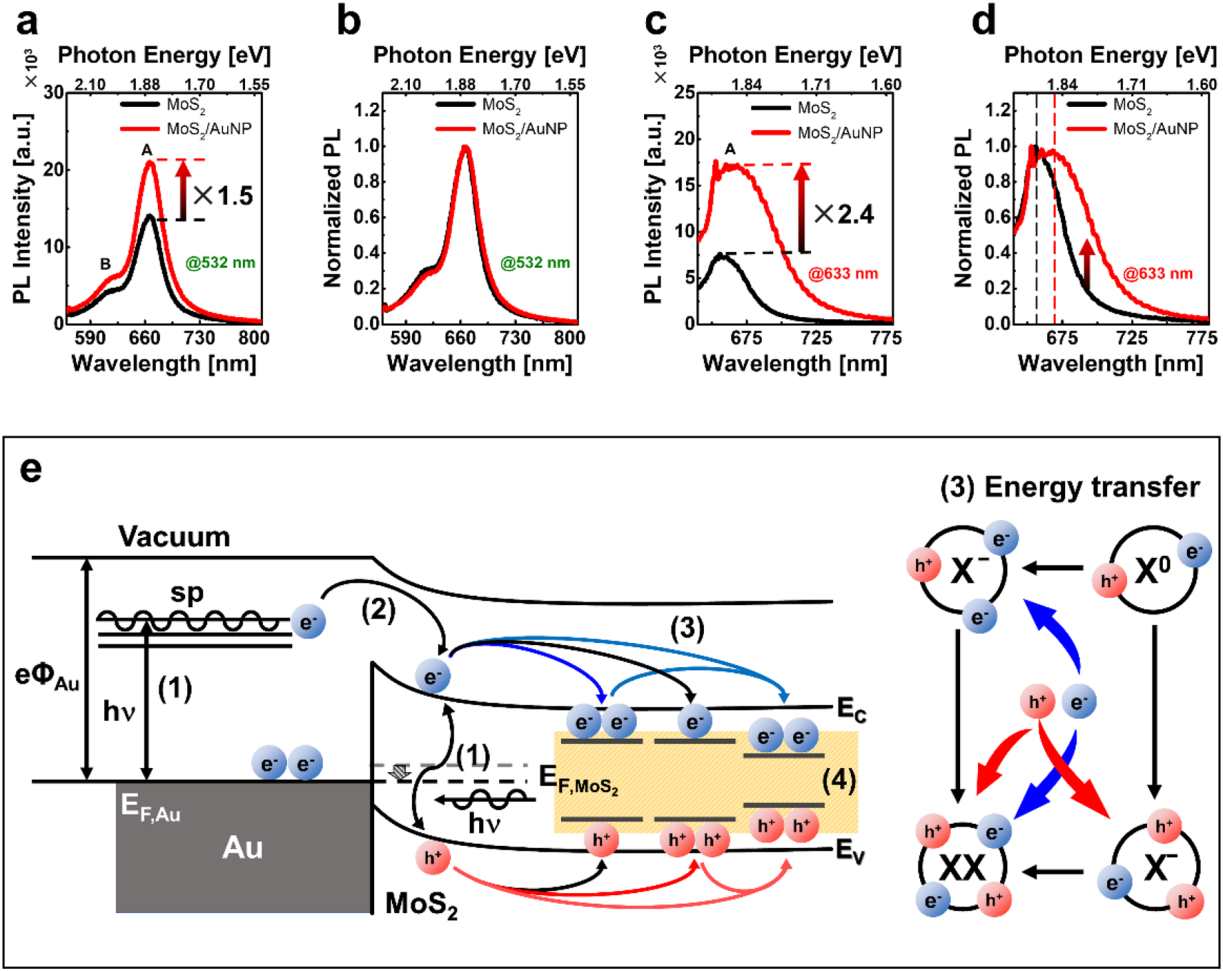
PL spectra of 532 nm and 632.8 nm excitation and mechanism of plasmon-exciton coupling. PL spectra for (**a**) 532 nm and (**c**) 632.8 nm excitation. Normalized PL spectra for (**b**) 532 nm and (**d**) 632.8 nm excitation. (**e**) Schematics of plasmon-exciton coupling mechanisms of each exciton mode on the MoS_2_/AuNP hybrid. (1) excitation process. (2) energy transfer. (3) recombination process. (4) EM field enhancement.

Figure 2a–d shows the PL spectra with 532 nm and 632.8 nm excitation to investigate the plasmonic response of the hybrid structure, respectively. The peak positions of the PL (denoted as A and B, respectively) are 1.90–1.95 eV and 2.05–2.10 eV, respectively, which are consistent with the previous results for monolayer MoS_2_^38^.

The peak intensity of the A exciton was enhanced about 1.5 and 2.4 times under 532 nm and 632.8 nm excitation, respectively. The normalized PL spectra with 532 nm excitation are shown in Fig. 2b. The normalized spectrum barely changed by the AuNPs, owing to the off-resonance excitation of the LSPRs.

In contrast, 632.8 nm excited PL spectra showed significant changes with peak positions and line width, as shown in Fig. 2d. The peak position of the spectrum is red-shifted, and the linewidth of the spectrum is broadened in MoS_2_/AuNP hybrid structure. The observed phenomena can be described by the complex interaction between AuNP and MoS_2_, such as plasmon-exciton coupling, local doping, strain, etc.

## Discussion

We suggest that the exciton-plasmon coupling scheme can explain the exciton recombination mechanism in the MoS_2_/AuNP hybrid structure. As depicted in Fig. 2e, the energy transfer mechanism was demonstrated considering selective exciton excitation in the MoS_2_/AuNP hybrid structure under the resonant excitation of LSPRs. The recombination of each exciton was followed by excitation, energy transfer, recombination, and EM field enhancement.

The first step of the recombination pathways in the MoS_2_/AuNP hybrid structure is the excitation process. The optically excited electron in the AuNP has radiative and nonradiative pathways in the relaxation processes. The nonradiative relaxation of the photoexcited carrier in AuNP, such as carrier-carrier scattering, is much faster than radiative electron–hole recombination^39^. The strength of nonradiative relaxation processes depends on the resonant plasmonic optical response of the AuNP. Also, the resonant energy transfer in nonradiative relaxation processes has to consider the metal–semiconductor energy band alignment in the plasmonic hybrid structure^40^. The condition of resonance energy transfer was estimated through absorption spectra and simulation, as mentioned in Fig. 1.

**Figure 3:**
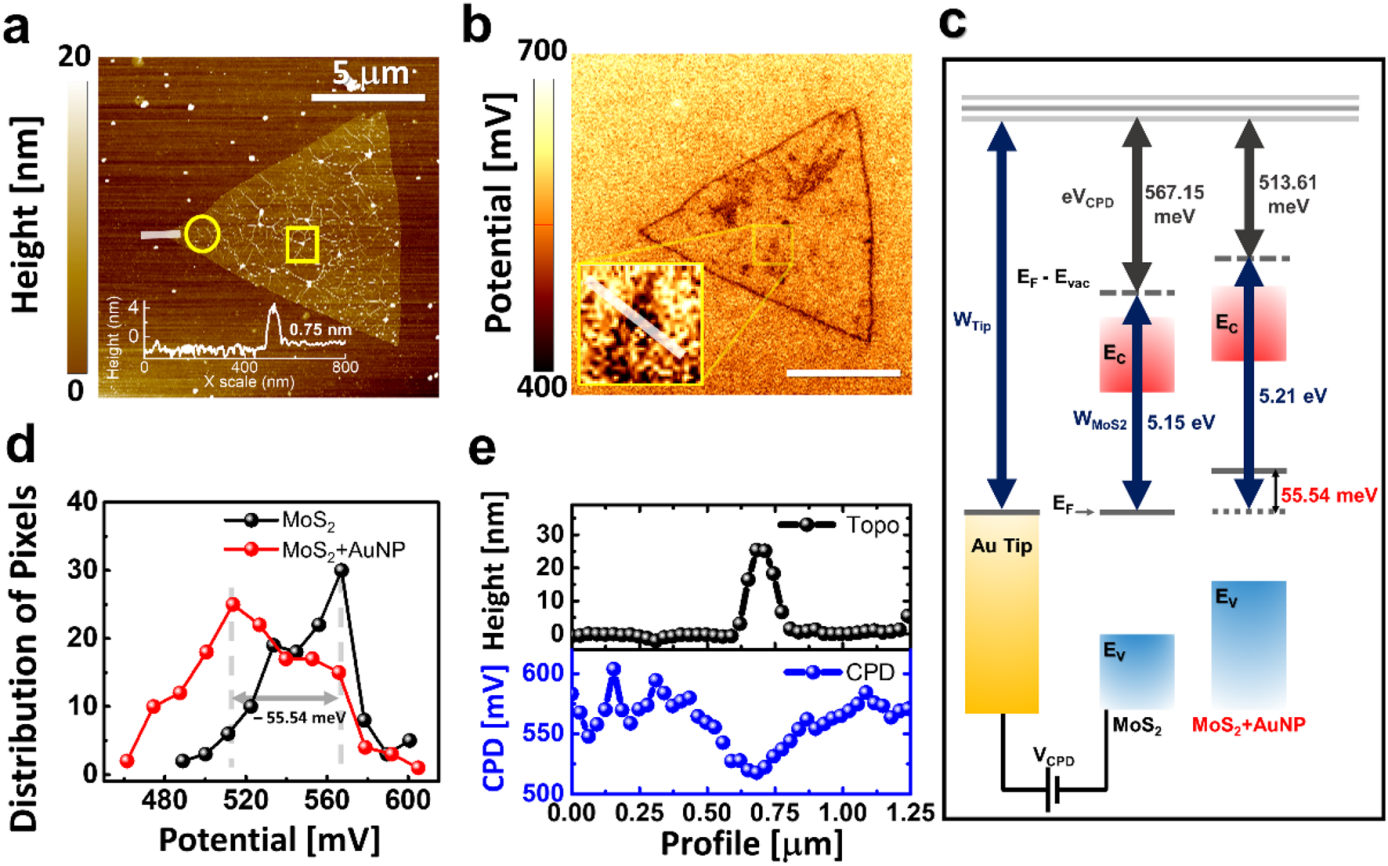
Scanning probe microscope results and potential diagram. (**a**) Topography image and line profile at the white line (inset). (**b**) KPFM result of electrostatic potentials (scale bar 5 μm). The inset shows the magnification of the MoS_2_/AuNP hybrid (size: 700 × 700 nm^2^). (**c**) Schematics of the energy band diagram. (**d**) Distribution of pixels in the KPFM result. (**e**) The line profile of topograph and CPD on the white line in the inset of (**b**).

The second step is the energy transfer between the AuNP and the monolayer MoS_2_. To investigate the energy transfer mechanism, PL emission and electrostatic characteristics were measured. The line width broadening in PL spectra implies the excessive charge density in the excitonic feature of the MoS_2_/AuNP hybrid structure under resonant excitation (Fig. 2a–d). Figure 3a,b show the topography and surface potential map of MoS_2_/AuNP plasmonic hybrid structures, respectively. The thickness of the MoS_2_ is ~ 0.75 nm on the edge of the monolayer MoS_2_. In addition, monolayer MoS_2_ film has wrinkles due to ~ 30 nm of the AuNP particles. Notably, the topography and potential images have different appearances on MoS_2_/AuNP (square) and bare MoS_2_. Besides, the surface, including flat and wrinkled areas without the nanoparticles (circle), does not make a distinct electrical potential difference.

As illustrated in Fig. 3c, the contact potential difference (eV_CPD_) on the MoS_2_ and hybrid structures is 567.15 meV and 513.61 meV, respectively. The equilibrium state of the Fermi level was p-doped due to the − 55.54 meV Fermi level modulation by contact on the AuNP and MoS_2_. The contact in the MoS_2_/AuNP hybrid structure was estimated as Ohmic contact due to the smaller work function of MoS_2_ (5.15 eV) than AuNP in the case of p-doped semiconductors^41–43^. Notably, the photo-excited carriers in AuNP and plasmonic hot-carriers moved toward the MoS_2_ film by the downward energy band bending at the interface^44^. The carrier injection in the interface can modulate the exciton bindings in the MoS_2_ film^45,46^.

Figure 3d,e show the electrical potential difference on the MoS_2_/AuNP plasmonic hybrid structure with the V_CPD_ distribution and profile. The difference of the eV_CPD_ between the monolayer MoS_2_ and Si/SiO_2_ substrate is − 50 meV. However, the distribution of the eV_CPD_ on the MoS_2_/AuNP structures was decreased to − 55.54 meV compared to the flat and wrinkled surface of MoS_2_. The result of the KPFM demonstrates that the monolayer MoS_2_/AuNP hybrid structure was p-doped, as mentioned in Raman spectra (Fig. 1c).

**Figure 4:**
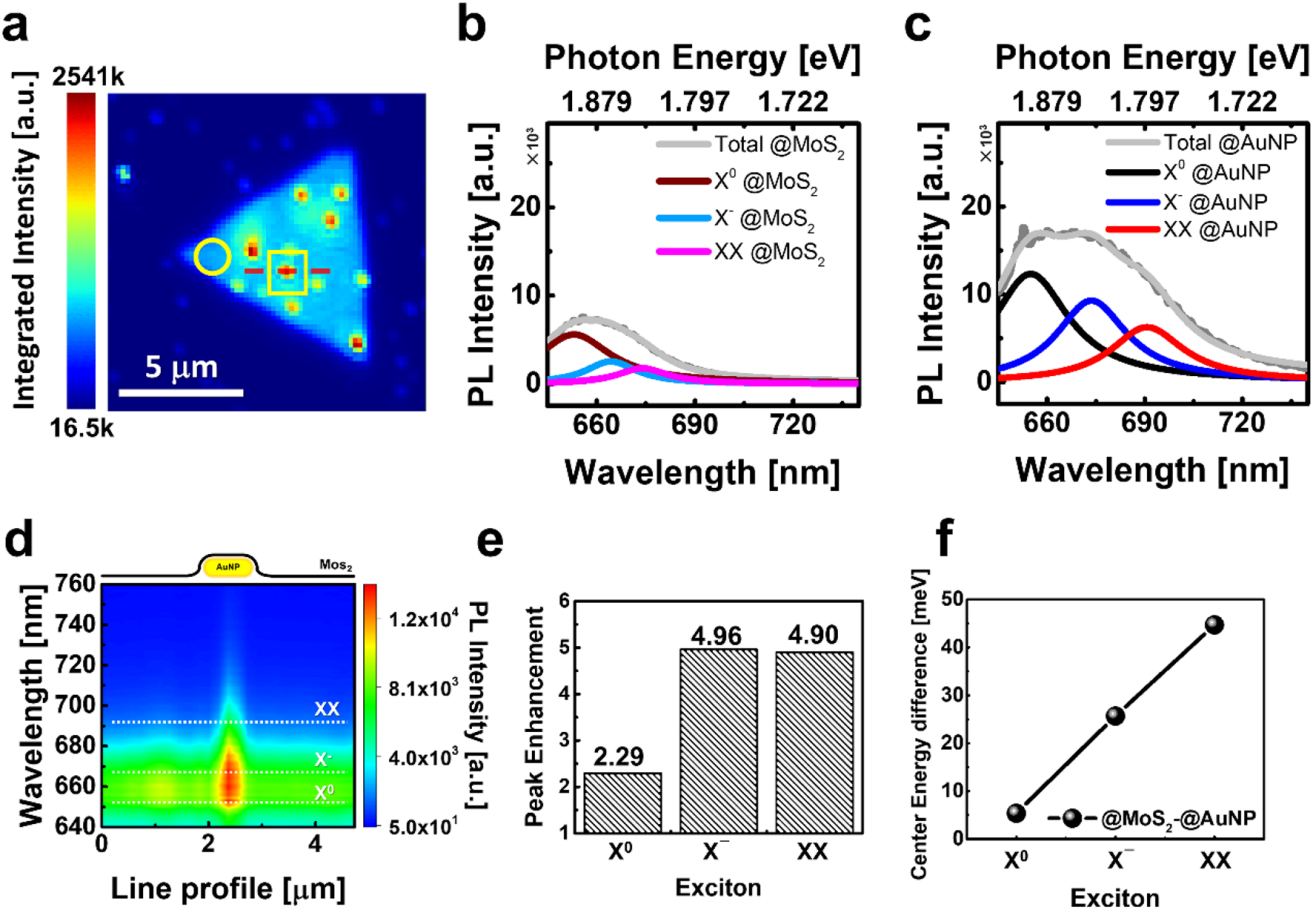
PL hyper spectra of 632.8 nm excitation and deconvolution results. (**a**) Integrated PL intensity mapping image. (Circle) Monolayer bare MoS_2_. (Square) Monolayer MoS_2_/AuNP hybrid. PL spectra and Lorentzian deconvolution results for each exciton in (**b**) the bare MoS_2_ and (**c**) the MoS_2_/AuNP hybrid. (**d**) The hyperspectral PL image on the red line in (**a**). (**e**) integrated intensity and (**f**) center energy from the result of deconvoluted PL spectra in (**b**) and (**c**).

The recombination process in each exciton mode was analyzed from the deconvoluted PL spectra. The energy relaxation in neutral exciton was recombinant to the trion and biexciton mode due to the plasmon-exciton coupling (Fig. 4). The interaction between MoS_2_ and the AuNP can be explained by introducing plasmonic coupling contributions in the excitation and emission processes of excitons. The majority of the PL enhancement was caused by induced local electric field enhancement at the interface between MoS_2_ and AuNP^47^. The total enhancement *g(ω)* on the plasmonic hybrid structures of the aggregated MoS_2_/AuNP is considered in two independent processes, excitation enhancement *g*_*exc*_*(ω*_*exc*_*)* and emission enhancement *g*_*em*_*(ω)* as following equations as^48^:

1 $$g\left( \omega \right) = \, g_{exc} \left( {\omega_{exc} } \right)g_{em} \left( \omega \right),$$

where ω is the frequency of the EM field, and *ω*_*exc*_ is the excitation laser frequency. The enhancement factor (EF) is complex to calculate due to the various exciton dynamics of the radiative/nonradiative decay rates and energy transfer. The energy transfer in the emission process is the response of interband charge transitions in the semiconductor and excited carrier transfer from the plasmonic structure. In addition, the local field enhancement depends on the polarization of the incident light according to the plasmonic response.

Moreover, the monolayer MoS_2_ has a strong absorption rate and PL emission spectra due to the weak dielectric screening and atomically thin spatial confinement of carriers. Neutral excitons (X^0^) in MoS_2_ can be coupled with the bound state of an electron and hole in the presence of residual excessive charge carriers under excitation. The charged neutral exciton of quasiparticles, called trions (X^–^), consists of two electrons and one hole. Also, the biexcitons (XX) are the formation of molecular states consisting of two excitons.

To estimate the plasmon-exciton coupling for each exciton, Fig. 4a–c show that the integrated PL intensity map and spectra in the MoS_2_ (circle) and MoS_2_/AuNP (square) under 200 µW at 632.8 nm excitations. The PL spectra were deconvoluted by Lorentzian formation for the intensity and center frequency estimation in each exciton mode (Fig. 4b–f)^26,49,50^. In Fig. 4b,c, the total PL peak intensities (gray) are 2.14 times higher in plasmonic hybrid structures than bare MoS_2_ film, as same in Fig. 2c,d. A significant enhancement in the 632.8 nm excitation was caused by the EM field resonance on the AuNP compared to the 532 nm excitation, as mentioned. Also, the change of PL intensity was barely observed on the natural wrinkles in the flake, which is consistent with the result in Fig. 3b. The linewidth broadening of the total PL spectra on the region of MoS_2_/AuNP can be explained by increasing contributions of the X^–^ and XX states.

Figure 4d shows the hyper spectral image of PL in the MoS_2_/AuNP hybrid structure along the red line in Fig. 4a. The observed spatially resolved PL intensity shows that the XX peak was clearly enhanced only at the MoS_2_/AuNP hybrid structure. Figure 4e shows the peak enhancement of each exciton mode, which results using the deconvolution of the PL spectra in the bare MoS_2_ and MoS_2_/AuNP hybrid structure. The enhancement of X^0^, X^–^, and XX is 2.29, 4.96, and 4.90 times, respectively. The intensities of X^–^ and XX are more than two times larger than X^0^ due to the large *g*_*exc*_*(ω*_*exc*_*)* of plasmon-exciton coupling and energy transfer.

Figure 4f shows the center energy difference in each exciton mode. The peak shift of neutral exciton is − 5.38 meV in MoS_2_/AuNP hybrid structure. Also, the difference of center frequency in X^–^ and XX is 25.68 meV and 44.69 meV, respectively. The peak shifts of X^–^ and XX are significantly larger than X^0^ because of the effectively local p-dopping by the AuNP applied local tensile strain and energy transfer in direct contact between nanoparticle and MoS_2_. It is observed that the broadening of the peak (see supplementary information S1) denotes the increase of the recombination rate, which also supports the existence of the local p-doping effect and the effective charge transfer from metal nanoparticles to the MoS_2_ film.

The analyzed each exciton mode from PL spectra under 632.8 nm excitation shows the plasmon-exciton coupling and energy transfer mechanism in MoS_2_/AuNP plasmonic hybrid structure^1,51,52^. The photoexcited carrier in AuNP was coupled with neutral exciton in MoS_2_. Besides, effectively p-doped MoS_2_ due to the induced local tensile strain of AuNP creates the hole charge in the MoS_2_ semiconductor. Therefore, the enhancement mechanism in X^–^ and XX can be verified by energy transfer and plasmon-exciton coupling in the MoS_2_/AuNP hybrid structure under the excitation of resonance condition of the AuNP.

**Figure 5:**
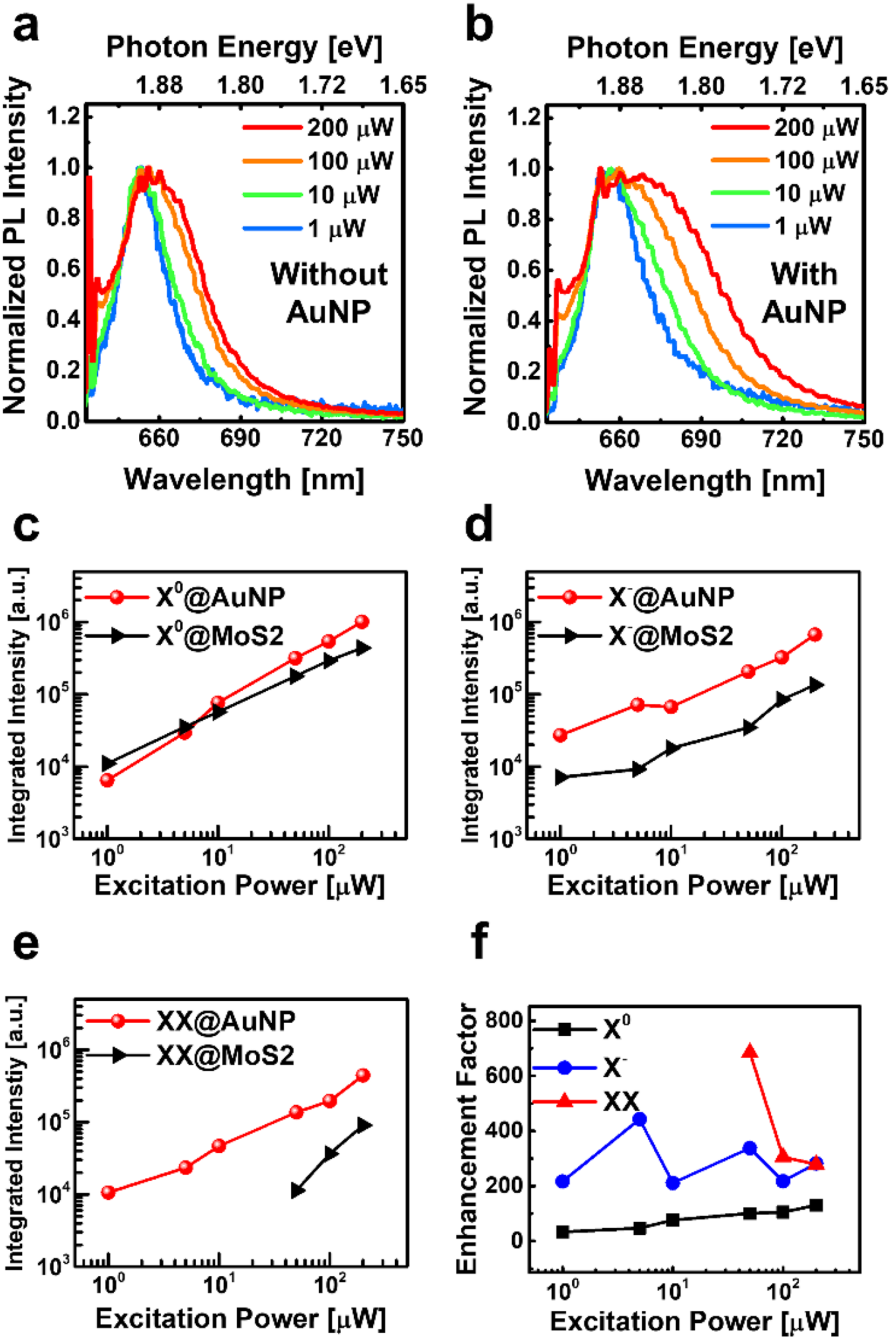
Fundamental power dependence normalized PL spectra and deconvolution results. Power dependence Normalized PL spectra in (**a**) the monolayer bare MoS_2_ and (**b**) the MoS_2_/AuNP hybrid structure. The integrated intensity of deconvolution results for (**c**) neutral exciton (X^0^), (**d**) trion (X^–^), and (**e**) biexciton (XX), respectively. (**f**) Calculated enhancement factor of each exciton mode.

To tell the optical doping and plasmon-exciton coupling, we measured the excitation power-dependent PL spectra in the monolayer MoS_2_/AuNP hybrid structure (Fig. 5). To distinguish the optical doping effects and plasmon-exciton coupling, power-dependent PL spectra of MoS_2_ without and with AuNP were shown in Fig. 5a,b, respectively. The observed PL spectra of MoS_2_ with/without AuNP were broadened and red-shifted when excitation power was higher than 100 μW. It can be explained by the thermal exciton-phonon coupling and optical doping effect, as reported elsewhere^37,53^.

The integrated intensity of X^0^, X^–^, and XX as a function of excitation power is shown in Fig. 5c–e, respectively. The X^0^ is not drastically enhanced, but the integrated intensity of the X^–^ was enhanced about 3.8–7.8 times. Although the peak intensity of the XX was hardly detectable lower than 10 μW excitations in bare MoS_2_, XX peaks appeared on the MoS_2_/AuNP hybrid structure.

The EF of each exciton spectra is shown in Fig. 5f. It was calculated by the following equation^54^:

2 $$EF=\frac{{I}_{Plasmonic}}{{I}_{Bare}}\times \frac{{A}_{Bare}}{{A}_{Plasmonic}},$$

where the ${I}_{Plasmonic}$ and the ${I}_{Bare}$ refer to the exciton peak intensity in plasmonic hybrid structures and the bare MoS_2_ film, respectively. The area of *A*_*Bare*_ (1 µm^2^) and *A*_*Plasmonic*_ (0.0176 µm^2^) is determined by the excitation laser spot and EM field enhancement area in the plasmonic structure, respectively.

The calculated enhancement factor of the X^0^, X^–^, and XX is ~ 100, ~ 300, and over 300, respectively. The enhancement factor can be calculated over 10 μW excitation power due to the absence of the XX signal in bare MoS_2_, as shown in Fig. 5f (red). The reason is that the high enhancement factor in MoS_2_/AuNP hybrid structure is the result of the strong electric field confinement originating from the LSPR coupling in AuNP. The optical response of the XX is the optical doping dependence excitonic features, as mentioned in the previous papers^26,55^.

**Figure 6:**
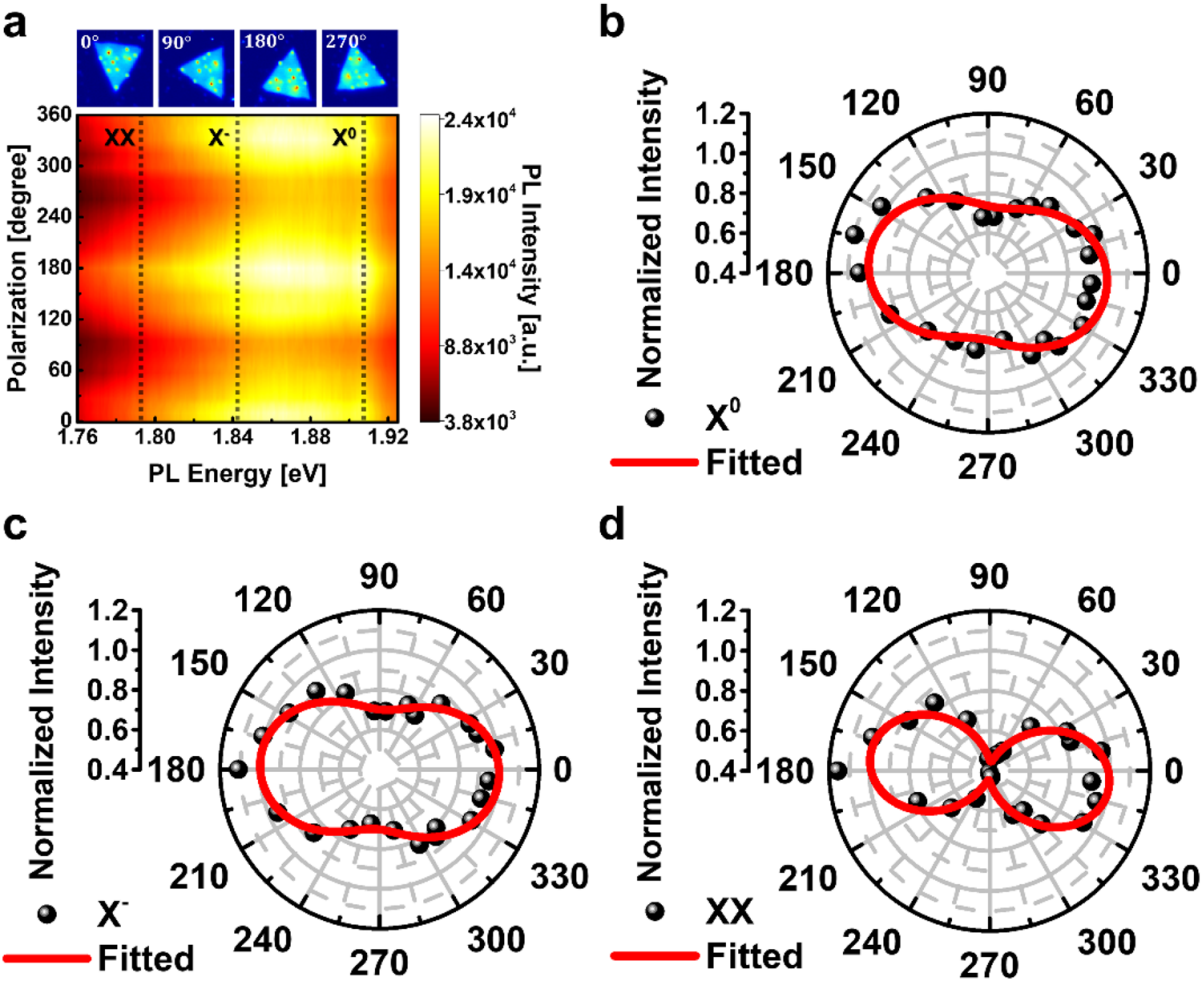
Polarization dependency of each exciton mode in the MoS_2_/AuNP hybrid. (**a**) Polarization-dependent hyperspectral PL image in the MoS_2_/AuNP hybrid. The normalized intensity of deconvoluted PL spectra depends on the excitation polarization for (**b**) the X^0^, (**c**) X^–^, and (**d**) XX modes.

To investigate the role of anisotropy in the optical response of the non-centrosymmetric AuNP and MoS_2_ hybrid structure, Fig. 6a shows the polarization-resolved PL spectra. The sample structure was rotated to avoid any unwanted optical misalignment to control the incident polarization angle *θ*. The periodic intensity variations of the PL spectra imply that the MoS_2_ excitons strongly correlated with polarization-dependent LSPRs coupling in anisotropic resonators.

The quantified normalized intensities of each exciton are shown in Fig. 6b–d. The orientation of maximum optical response in plasmon-exciton coupling was calculated by the function of *I*_*0*_ + *I*_*1*_* cos*^*2*^* (θ—θ*_*max*_*)*, where *I*_*0*_ and *I*_*1*_ are constants of normalized intensity, and *θ*_*max*_ is the angle at the maximum intensity by deconvolution of the Lorentzian function.

In previous studies, the general bare MoS_2_ film does not correlate with the incident angle of linear polarization *θ* due to high lattice symmetry^56,57^. However, the results of the polarization-resolved PL spectra for each exciton in the MoS_2_/AuNP hybrid structure demonstrate anisotropy of the optical response as a function of the incident orientation. The polarizability (*I*_*1*_) in X^0^ and X^–^ are 0.26 and 0.30, respectively, but XX is 0.55. It is the nature of the plasmonic response that local electric field enhancement is relevant not only to excitation polarization but also to emission spectral range. This result agrees with the larger enhancement for XX than X^0^, again confirming that the polarization-sensitive plasmon excitation is responsible for the pronounced XX generation.

In conclusion, we investigated the enhanced plasmon-exciton coupling of X^0^, X^–^, and XX modes on non-centrosymmetric AuNP and monolayer MoS_2_ hybrid structure using absorption, Raman, PL spectra depending on the excitation wavelength, incident power, and polarization control. We explained the mechanism of plasmon-exciton coupling in each exciton mode, of which recombination pathways are followed in excitation, energy transfer, recombination, and EM field enhancement. The hybrid structure of specific plasmonic AuNP and TMDCs showed polarization-dependent optical responses and enhancement. Plasmon-biexciton coupling revealed higher polarizabilities than X^0^ and X^–^ because of optical doping-dependent excitonic features and plasmonic resonance. We believe that another degree of freedom to control and engineer the excitonic response provides a new pavement toward the development of optoelectronic nanodevices with TMDCs and supports the plasmonic application of innovative optoelectronic technology.

## Methods

### Monolayer MoS_2_ synthesis

Triangular-shaped MoS_2_ growth was carried out in an atmospheric pressure chemical vapor deposition. As Mo precursor, 0.3 g of ammonium heptamolybdate (AHM, Sigma-Aldrich, 431346) was dissolved into distilled (DI) water. The AHM solution was again mixed with NaOH solution (0.125 mol) and 0.2 ml of iodixanol solution (Sigma-Aldrich, Opti Prep, D1556) in the ratio of 0.3:2:0.2. The solution was spin-coated onto O_2_ plasma-treated substrate (300 nm oxide Si wafer), forming a uniform Mo-Na-C precursor matrix. Spinning condition and plasma powers are 4000 rpm for 40 s and 30 W for 1 min, respectively. Then, the substrate was cut into 1 × 1 cm^2^, loaded in zone 2 (outlet) with 0.2 g of S (Sigma-Aldrich, 212392) in zone 1 (inlet). For MoS_2_ growth, the tube furnace was ramped to 190 °C and 780 °C for S (zone 1) and Mo (zone 2). 300 sccm of N_2_ was injected for the ramping process (12 min), followed by the growth process with an increased flow rate (1000 sccm, 7 min).

### Plasmonic structure synthesis

The resulting split Au nano-rings could be obtained, followed by our previous research (Nano Lett. 2020, 20, 10, 7774–7782)^21^. Briefly, Au nano-prisms were employed as starting material en route to synthesize split Au nano-rings. First, vertices of Au nano-prisms were etched to thin Au nano-disks (~ 10 ± 1 nm in height) using Au^3+^ ion as an etchant. Subsequently, thin Au nano-disks were converted to Au nano-hexagons through depositing Au followed by an etching step, leading to thick Au nano-disks with a height of ~ 25 ± 2 nm. In the selective edge deposition of Pt, Pt atoms were decorated at the periphery of thick Au nano-disks in part aided by the presence of high-index facets that can reduce the activation energy barrier for Pt nucleation leading to Au@Pt disks. In the next step, the core Au domains were etched away by adding Au^3+^ ions resulting in the formation of split Pt nano-rings. Eventually, in the Au regrowth step, Au atoms were homogeneously reduced on the entire surface of split Pt nano-rings, leading to split Au nano-rings.

### Sample characterizations

An absorption hyper spectra map was obtained to analyze the optical response of the nano-ring/MoS_2_ structure. The measurement position was controlled with the piezo sample stage (PI, P-611K020 NanoCube) for precise positioning on the commercial inverted microscope (Nikon, ECLIPSE Ti-U). A broadband illumination of light (Nikon, D-LH/LC, color temperature: 3300 K) was used, and the beam was focused on the sample by 100 × objective (Nikon, CFI LU PLAN EPI, NA 0.80, WD 3.5 mm). The reflected optical signal was collected and recorded by the spectrometer (Princeton Instruments, SpectraPro 2300i, 150 lpmm, CCD camera) with sample position. The acquisition time for each point was 100 ms. The reference of reflectance spectra was set as on the bare Si/SiO_2_ surface under broadband tungsten lamp illumination.

The photoluminescence and Raman spectra were excited by 532 nm DPSS laser (Optoelectronicstech, MGL-III-532) and 632.8 nm He–Ne laser (Thorlabs, HNL210LB) with less than 200 µW after objective lens. For tight focusing, the single-mode fiber (Thorlabs, SM450) was used as a spatial mode filter. The polarization dependence experiment was performed by rotating the sample. PL and Raman hyper-spectra map was obtained with each 15-degree clockwise rotation of the sample.

The Lorentzian function was adapted to decompose each spectral peak for X^0^, X^–^, XX, B^0^ excitons in PL and E^1^_2g_, A_1g_ peak positions in Raman spectra using Python (Ver 3.7.4, Scipy module). To investigate the surface potential dependency, KPFM and topography were acquired simultaneously by using a commercial scanning probe microscope (Parksystems, XE-NSOM) with an Au-coated cantilever (MikroMasch, NSC-14-Cr-Au).

### Finite element method simulation

For calculating the optical response of a gold nanoparticle, commercially-available software (COMSOL Multiphysics, V6.0) was utilized. All simulations were performed in 3D with Wave Optics Module. The simulation area consists of two domains: core and shell. The core domain comprises a nanoparticle and surrounding medium, and the shell domain is composed of perfectly matched layers (PML). In addition, the scattering boundary condition was applied at the outer boundary of the shell domain. In the simulations, all geometric parameters were obtained from SEM images, as shown in Fig. 1b. The complex refractive index of gold was taken from the literature^58^. In addition, the effective permittivity of the surrounding medium was assumed to be 1.58. To calculate the absorbance spectrum of the gold nanoparticles, an absorption cross-section (${\sigma }_{abs}$) was calculated. ${\sigma }_{abs}$ can be defined as ${\sigma }_{abs}={W}_{abs}/{P}_{inc},$ where ${P}_{inc}$ and ${W}_{abs}$ are incident irradiance and energy rate absorbed by gold nanoparticles, respectively.

## Supplementary Information

Supplementary Information.

## Data Availability

All data generated and the datasets used and analyzed during the current study are available from the corresponding author on reasonable request.
