# Supplementary material for: Plasmon-exciton couplings in the MoS2/AuNP plasmonic hybrid structure
Source: Sci Rep. 2022 Dec 23;12:22252. doi: 10.1038/s41598-022-26485-4 (PMC9789063; doi:10.1038/s41598-022-26485-4)
Supplement: Supplementary file 1 — Supplementary Information. [file 41598_2022_26485_MOESM1_ESM.pdf]

Supplementary Information

Plasmon-exciton couplings in the MoS_2/AuNP plasmonic hybrid structure

Hyuntae Kim[1 †], Jaeseung Im[1 †], Kiin Nam[1], Gang Hee Han[1], Jin Young Park[1], Sung Jae Yoo[2], MohammadNavid Haddadnezhad[2], Sungho Park[2], Woongkyu Park[3], Jae Sung Ahn[3], Doojae Park[4], Mun Seok Jeong[5], and Soobong Choi[1]*

[1]Department of Physics, Incheon National University, Incheon 22012, Republic of Korea

[2]Department of Chemistry, Sungkyunkwan University, Suwon 16419, Republic of Korea

[3]Medical & Bio Photonics Research Center, Korea Photonics Technology Institute (KOPTI), Gwangju 61007, Republic of Korea

[4]Department of Applied Optics and Physics, Hallym University, Chuncheon 24252, Republic of Korea

[5]Department of Physics, Department of Energy Engineering, Hanyang University, Seoul 04763, Republic of Korea

[*]Correspondence: Soobong Choi (sbchoi@inu.ac.kr)

[†]Author contribution: Hyuntae Kim and Jaeseung Im are contributed equally.

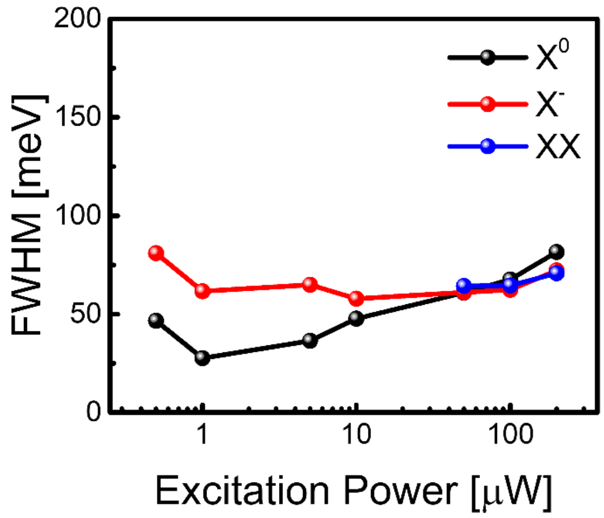

Figure S1. The full width at half maximum (FWHM) of deconvoluted photoluminescence (figure 5 in the main text) as a function of excitation power.
